# Cross-Reactivity of Peanut Allergens

**DOI:** 10.1007/s11882-014-0426-8

**Published:** 2014-02-20

**Authors:** Merima Bublin, Heimo Breiteneder

**Affiliations:** Department of Pathophysiology and Allergy Research, Medical University of Vienna, Währinger Gürtel 18-20, 1090 Vienna, Austria

**Keywords:** 2S albumin, Allergy, Allergen, Ara h 1, Ara h 2, Ara h 3, Ara h 8, Ara h 9, Bet v 1, Cross-reactivity, Cupin, Food allergy, Food allergen, Immunoglobulin E, Lentil allergy, Lupin allergy, NSLTP, Oleosins, Protein family, Peanut, Peanut allergen, Profilin, Prolamin, Soy allergy, Tree nut allergy

## Abstract

Peanut seeds are currently widely used as source of human food ingredients in the United States of America and in European countries due to their high quality protein and oil content. This article describes the classification and molecular biology of peanut seed allergens with particular reference to their cross-reactivities. Currently, the IUIS allergen nomenclature subcommittee accepts 12 peanut allergens. Two allergens belong to the cupin and four to the prolamin superfamily, and six are distributed among profilins, Bet v 1-like proteins, oleosins, and defensins. Clinical observations frequently report an association of peanut allergy with allergies to legumes, tree nuts, seeds, fruits and pollen. Molecular cross-reactivity has been described between members of the Bet v 1-like proteins, the non-specific lipid transfer proteins, and the profilins. This review also addresses the less well-studied cross-reactivity between cupin and prolamin allergens of peanuts and of other plant food sources and the recently discovered cross-reactivity between peanut allergens of unrelated protein families.

## Introduction

Peanuts are the seeds of the peanut plant (*Arachis hypogaea*) which is a member of the legume family (Fabaceae). The peanut is botanically related to beans and peas but not to tree nuts. The typical peanut seed pod which usually contains two seeds matures buried underground. Peanuts are very rich in nutrients and are one of the basic crops of India, China, the USA and West Africa. Peanuts contain 44–56 % oil and 22–30 % protein [1]. The total protein content of three of the most commonly used peanut cultivars (Valencia, Virginia, and Spanish) was determined to be between 24 and 29 % [2]. Most of the protein content is made up by seed storage proteins of the cupin or prolamin superfamilies. The cupin Ara h 1 was determined to contribute 12–16 %, and the 2S albumin Ara h 2 5.9–9.3 % to the total protein content of a peanut [2]. In a recent study, all known peanut allergen classes were determined to comprise 85 % of the total protein content of peanut while Ara h 1, Ara h 2, and Ara h 3 together accounted for 75 % [3].

### Foundation of Cross-Reactivity

Cross-reactivity relies on the presence of conserved antibody-accessible surface structures of proteins and is hence observed in general between members of the same protein family. Protein evolution is linked to the evolution of species. Thus proteins that appeared very early in the evolutionary process are distributed much wider than proteins that appeared later on. The cupin and the Bet v 1 architectures can be traced back to the Archaea. Archaea are also the oldest form of life on earth with an estimated age of 3.5 billion years [4, 5]. The typical cupin domain first appeared in extremophile Archaea [6]. The most important storage proteins of legumes, tree nuts and seeds are members of the cupin superfamily [7]. *Aeropyrum pernix*, an archaeon that was isolated from a hydrothermal vent near a Japanese island in 1996 [8], produces the protein APE2225 (PDB accession no. 2NS9) whose architecture is identical to that of Bet v 1 (PDB 1BV1). Like members of the cupin superfamily, Bet v 1-like proteins can be found in all three domains of life including birch pollen and peanut [9]. In contrast, the prolamin superfamily seems to be of a later origin. Non-specific lipid transfer proteins (nsLTPs) have only been identified in seed plants but are not even present in algae [10•]. Thus, protein evolution explains the occurrence of so-called panallergens - such as the Bet v 1 homologs — in many unrelated allergen sources. However, the in vitro-observed IgE cross-reactivity of such panallergens is not always associated with clinical symptoms. Only recently, cross-reactivity between two unrelated protein architectures has been described for the first time. The cross-reactivity between the 2S albumin Ara h 2 and the cupins Ara h 1 and 3 is based on the presence of short similar structural motifs [11•].

### Clinical Relevance of Cross-Reactivity

**Fig. 1:**
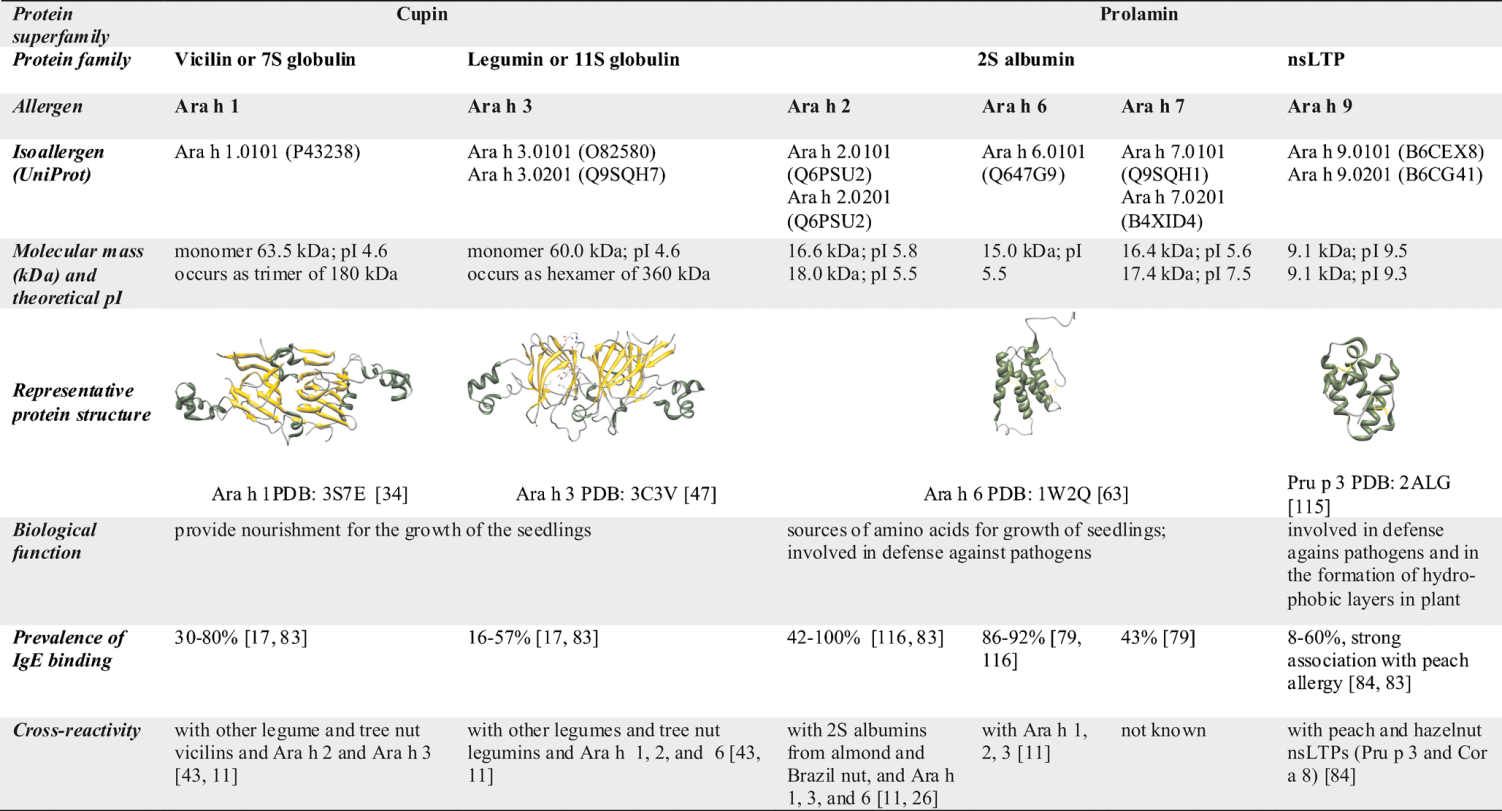
Allergenic cupins and prolamins from peanut

**Fig. 2:**
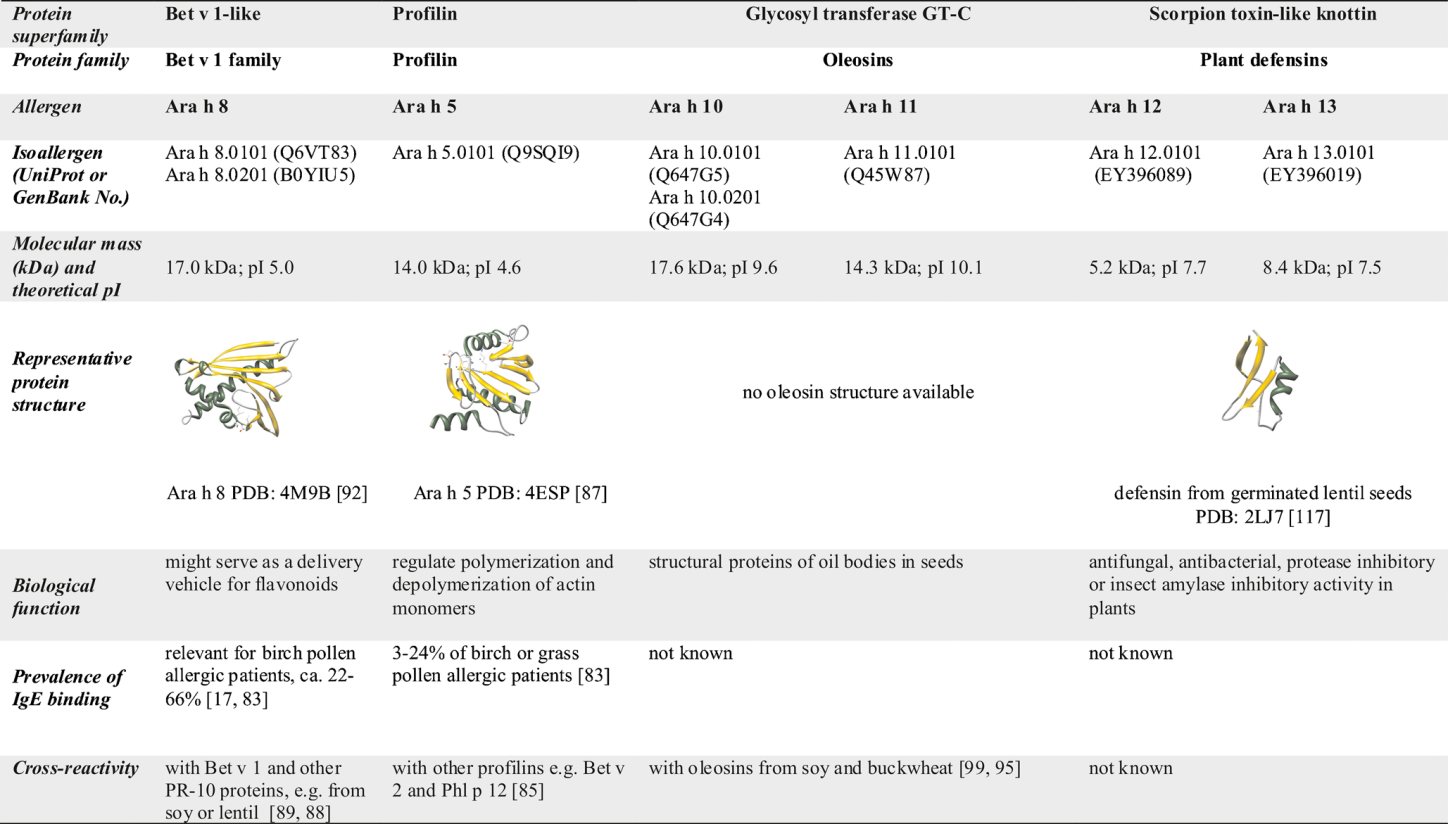
Peanut allergens from the Bet v 1, profilin, oleosin, and defensin families

Twelve allergens of peanut have been included in the official allergen nomenclature database (http://www.allergen.org/) to date. They belong to the cupin (Ara h 1, Ara h 3), the prolamin (Ara h 2, Ara h 6, Ara h 7, Ara h 9), the profilin (Ara h 5), the Bet v 1 (Ara h 8), the glycosyl transferase GT-C (Ara h 10, Ara h 11), and the scorpion toxin-like knottin (Ara h 12, Ara h 13) superfamilies (Figs. 1 and 2).

Ara h 8, a homolog of the major birch pollen allergen Bet v 1, and Ara h 5, a profilin, are mostly involved in pollen-associated food allergy, while the peanut nsLTP Ara h 9 is involved in the so-called nsLTP-syndrome due to the cross-reactivity with their homologs in birch and/or grass pollen or in fruits and seeds, respectively. Ara h 2 was described as the most important peanut allergen, as it was identified as a predictor of clinical reactivity to peanut [12–14]. On the other hand, monosensitization to a single peanut allergen is relatively rare [15], and patients with monosensitization to Ara h 2 had a significantly lower symptoms severity score than polysensitized subjects and a lower level of allergen-specific IgE against peanut extract and Ara h 2 [16]. Polysensitization to Ara h 2 and Ara h 1 and/or Ara h 3 appeared to be predictive of more severe reactions [16–19].

In peanut-allergic patients a clinically relevant sensitization to other legumes such as soybean, lupin, lentil, or pea occurs; however, little information is available. In a group of 39 peanut-sensitized patients, 82, 55, and 87 % of patients were also sensitized to lupine, pea, and soybean, respectively, whereas, based on DBPCFC, 29–35 % had symptoms to these beans [20]. In a recent study, Klemens and colleagues showed that 60 % of soy-allergic patients had a concomitant peanut allergy and significantly more often specific IgE to soy extract, the soy 2S albumin Gly m 8 and the soy cupins Gly m 5 and Gly m 6 compared to the peanut-tolerant group [21•].

Between 20 and 40 % of peanut-allergic individuals have a co-existing allergy to taxonomically distantly related tree nuts [22, 23]. In a large study including 324 peanut-allergic patients, 86 % were sensitized to tree nuts, and 34 % had clinical documented allergy [24]. The numbers may even be higher than those reported in a study by Glaspole and colleagues where up to 60 % of peanut-allergic individuals examined in their adult allergy clinic were allergic to one or more tree nuts with the most common reactivity of 49 % to hazelnuts [25].

In the majority of the cases, the identity of the cross-reactive allergens was not investigated.

Using nut protein extracts and sera from subjects with peanut and tree nut allergy in inhibition ELISA, de Leon and colleagues demonstrated serum IgE cross-reactivity between allergens present in peanut, almond, Brazil nut and hazelnut [26]. No IgE cross-reactivity between peanut and cashew was detected. In a next step, de Leon and co-workers purified peanut-specific IgE from sera of two peanut-allergic individuals [27]. They then isolated basophils from atopic but non-peanut-allergic subjects, stripped the cells of bound IgE and resensitized them with purified peanut-specific IgE. The basophils were activated by extracts from peanut, almond, Brazil nut, and hazelnut but to a lesser degree by cashew.

In vivo cross-reactivity between cashew, walnut and peanut was assessed in C3H/HeJ mice [28]. Mice were sensitized by intraperitoneal injection of protein extracts from cashew alone or from cashew and walnut. Mice sensitized to only cashew reacted almost as strongly to walnut as they did to cashew, with the vicilin-like Jug r 2 being implicated as the cross-reactive allergen. Only mild symptoms were observed when challenging these mice with peanut. Mice sensitized to cashew and walnut had higher anaphylactic scores when challenged with the tree nuts but still produced weaker reactions to peanut. Both groups of mice showed strong T cell proliferative responses to cashew and walnut and weaker responses to peanut.

## Allergenic Peanut Cupins

A proteomic study by Chassaigne and colleagues revealed the presence of two Ara h 1 and six Ara h 3 isoforms in protein extracts of the peanut variety Virginia [29]. In this study, Ara h 1 was resolved into ten and Ara h 3 into eight protein spots illustrating the complexity of the peanut allergome. Although the number of proteins in the peanut seed proteome is relatively low, the presence of the numerous isoforms for each protein family member complicates the proteomic investigation [3]. Furthermore, Ara h 1 and Ara h 3 are incorporated into high molecular weight protein complexes upon roasting [30].

### Ara h 1

The cupin allergen Ara h 1 has been identified as member of the vicilin seed storage family [31]. The vicilins or 7S globulins are typically disk-shaped trimeric proteins whose subunit compositions vary considerably due to the extent of post-translational proteolytic processing and glycosylation [32]. They lack cysteine residues and hence cannot form disulfide bonds. The crystal structure of Ara h 1 has been elucidated and can be accessed under the PDB (protein database, http://www.rcsb.org/) numbers 3SMH [33] and 3S7I [34•] (Fig. 1). Besides Ara h 1, a range of allergenic vicilins has been described, including those from soybean (Gly m 5) [35], pea (Pis s 1) [36], cashew nut (Ana o 1) [37], walnut (Jug r 2) [38], lentil (Len c 1) [39], sesame (Ses i 3) [40], and hazelnut (Cor a 11) [41]. A crystal structure for the Ara h 1-related allergen Gly m 5 homotrimer is available [42]. Barre and colleagues built models of vicilin-like allergens from peanut (Ara h 1), walnut (Jug r 2), hazelnut (Cor a 11), and cashew nut (Ana o 1) using the x-ray coordinates of soybean Gly m 5 to illustrate the structural similarity of these allergens [43]. In addition, their molecular modeling study included one experiment with a pool of an undisclosed number of sera from peanut-allergic patients, which indicated IgE reactivity to the tree nut allergens Ana o 1, Cor a 11, and Jug r 2, as well as to the soybean allergen, Gly m 5.

The question of allergenic potential of cross-reactive 7S globulins and thus of the origin of sensitization was addressed in an interesting recent study using Brown Norway (BN) rats [44•]. BN rats were sensitized i.p. without the use of an adjuvant with purified 7S globulins from peanut (Ara h 1), hazelnut (Cor a 11), pea (Pis s 1), and soy (Gly m 5). Although the four related 7S globulins induced very similar IgG and IgE titers, Ara h 1 induced IgE of higher avidity than Cor a 11 and Pis s 1, which had higher avidity than Gly m 5. Glaspole and colleagues reported that hazelnut-specific T cell lines obtained from patients with co-allergy to hazelnut and peanut proliferated upon stimulation with Ara h 1 or Ara h 2 and expressed both IL-5 and INF-γ [25]. Shared T cell epitopes between hazelnut and peanut proteins may contribute to the observed co-sensitization that was previously also observed on the IgE level [26].

A recent study used peptide arrays to examine IgE-binding to sequential B cell epitopes of Ara h 1 and the related walnut allergen Jug r 2 from sera of 32 peanut-allergic patients with or without clinically relevant walnut allergy [45]. Interestingly, no differences in the recognition of these peptides were observed between the two groups of patients nor could any relevant cross-reactive IgE antibodies be detected in inhibition assays. The authors concluded that the sequence stretches previously identified as sequential IgE-binding epitopes of Ara h 1, Ara h 2, and Ara h 3 had no equivalents in walnut allergens.

### Ara h 3

Ara h 3 is a cupin allergen identified in peanut that belongs to the legumin family [46]. The allergen nomenclature subcommittee has renamed Ara h 4.01 to Ara h 3.02 as its sequence shares 91 % identity with that of Ara h 3, by far exceeding the 67 % identity threshold for naming isoallergens [7]. Legumins or 11S globulins are hexameric proteins that are found in the seeds of many plants. Their subunits are synthesized as a single polypeptide which is then cleaved to give rise to an acidic and a basic polypeptide chain that are linked by a single disulfide bond [32]. Legumins are not usually glycosylated. The crystal structure of Ara h 3 was determined and can be accessed under the PDB number 3C3V [47] (Fig. 1). Additional allergenic legumins have been described in legumes, tree nuts and seeds including those from soybean (Gly m 6) [48], cashew nut (Ana o 2) [49], walnut (Jug r 4) [50], sesame (Ses i 7) [51], and hazelnut (Cor a 9) [52]. Crystal structures for Ara h 3-related allergens are available for Gly m 6.0101 [53] and Gly m 6.0501 [54, 55] from soybean and for Pru du 6 from almond [56].

Barre and coworkers mapped linear IgE epitopes identified in Ara h 3, Jug r 4, Cor a 9, and Ana o 2 on three-dimensional models of these allergens built by homology modeling using the Gly m 6 structure 1OD5 from Adachi as a template [57]. The authors’ analyses revealed that these surface-exposed IgE epitopes exhibited some structural similarities, thus accounting for the observed IgE cross-reactivity between peanut and tree nut allergens. The legumin-like Sin a 2 has been identified as a diagnostic marker to indicate a risk for severe symptoms triggered by mustard consumption [58•]. Inhibition experiments using sera from mustard-allergic and Sin a 2-positive patients revealed IgE cross-reactivity between Sin a 1 and 11S globulins from peanut and tree nuts [59].

## Allergenic Peanut Prolamins

The prolamin superfamily contains the largest number of allergenic plant proteins [60]. It comprises several related protein families that contain a characteristic well-conserved pattern of eight cysteine residues but have low or almost no sequence identity. Despite the diversity of their function and low overall sequence identity between the conserved regions, these proteins all share a similar 3-dimensional structure consisting of bundles of four alpha-helices stabilized by disulfide bonds [61]. Members of the prolamin superfamily identified in peanut as allergens include 2S albumins and a type 1 non-specific lipid binding protein (nsLTP). Three peanut allergens belong to the 2S albumin family: Ara h 2, Ara h 6, and Ara h 7. As Ara h 2 shows 59 % amino acid sequence identity to Ara h 6 and 42 % to Ara h 7, they are designated as three different allergens in accordance to recommendations of WHO/IUIS Allergen Nomenclature Subcommittee. The peanut allergen Ara h 9 is a member of the nsLTP family which is widely distributed among plants.

### The 2S Albumins Ara h 2 and Ara h 6

The majority of 2S albumins are synthesized as proproteins which are then proteolytically processed in the vacuole into two subunits with four disulfide bonds. In contrast, peanut 2S albumins are not post-translationally modified except for the formation of disulfide bonds. As a result, the proteins occur in peanut as single polypeptide chains. Only the so-called asparagine-containing form of Ara h 6 has been found to be processed into a large (9.2 kDa) and a small subunit (5.4 kDa) linked via two disulfide bonds [62]. The structures of Ara h 2 (PDB: 3OB4) and Ara h 6 (PDB: 1W2Q) resemble that of other 2S albumins such as Ric c 3 (PDB: 1PSY) from castor bean, SFA-8 (PDB: 1S6D) from sunflower or Ber e 1 (PDB: 2LVF) from Brazil nut showing a common fold of antiparallel bundles of four helices held together by disulfide bonds in a right-handed superhelix fold (Fig. 1). A non-structured loop of Ara h 2 connecting alpha-helices 3 and 4, known also as a hypervariable loop, is almost doubled in size compared to Ara h 6. In addition, compared to Ara h 2, Ara h 6 contains a fifth disulfide bond linking the C-terminus to the compact fold, which in Ara h 2 is flexible and without regular secondary-structure elements [63, 64].

Ara h 2 has found the highest attention as it is regarded as the most potent peanut allergen. There are two isoforms of Ara h 2, designated as Ara h 2.0101 and Ara h 2.0201, with molecular masses of 16.7 and 18 kDa, respectively [65, 66]. Ara h 2.0201 contains an insertion of 12 amino acids in the hypervariable region containing the immunodominant IgE epitope [67]. The molecular masses of the two isoforms determined by mass spectrometric analysis correspond to the molecular weights calculated from the encoding genes [68], suggesting that Ara h 2 is not glycosylated as previously reported [65]. Among ten identified linear IgE-binding epitopes, three were immunodominant and located in the exposed and structurally flexible regions in the folded protein [69]. These three IgE-binding epitopes have been recently defined as the peptide biomarkers for prediction of symptomatic peanut allergy [12] and have been found to be particularly responsible for the cross-reactivity between Ara h 2 and Ara h 1, Ara h 3, and Ara h 6 [11•]. The conformational structure of Ara h 2 and Ara h 6 plays a critical role in allergenicity. Disruption of disulfide bonds by complete reduction and S-alkylation of these allergens drastically alters their IgE-binding capacity and effector cell activity [70, 71]. Dreskin and colleagues have found that Ara h 2 and Ara h 6 together account for the majority of the effector activity of whole peanut extract [72, 73], and demonstrated that Ara h 2 and Ara h 6 are not only major elicitors of anaphylaxis but can also effectively desensitize peanut-allergic mice [74•].

Although, co-sensitization to peanut and tree nuts is common, and many 2S albumins are known as important allergens in tree nuts such as Jug r 1 from walnut, Ber e 1 from Brazil nut, Cor a 14 from hazelnut, and Car i 1 from pecan, there are almost no experimental data about cross-reactivity between Ara h 2 and these allergens. Only one study using three sera from peanut-allergic patients showed that rAra h 2 shares IgE-binding epitopes with almond and Brazil nut, but not with cashew and hazelnut allergens [75]. Maleki and colleagues examined the IgE cross-reactivity between walnut and peanut allergens by testing sera of walnut- and peanut-allergic sera for IgE-binding to peptides representing predicted walnut and known and predicted peanut allergen epitopes [76]. Interestingly, a predicted Jug r 2 epitope not only bound IgE from the sera but also inhibited IgE-binding to the 2S albumin allergen Ara h 2. Jug r 2 and Ara h 2 belong to two different protein families and share only about 13 % sequence identity.

### The 2S Albumin Ara h 7

Initially, Ara h 7 was identified as an IgE-binding protein by phage display technology. In contrast to the conserved cysteine skeleton of 2S albumins with at least eight cysteines, Ara h 7.0101 had only six and missed two such residues in its C-terminus [77]. Schmidt and colleagues cloned a new isoform, Ara h 7.0201, with the typical 2S albumin cysteine signature, and they found that the determined amino acid sequence of Ara h 7.0101 was the consequence of a nucleotide insertion that resulted in a shift of the reading frame and the generation of a different C-terminus [78]. Accordingly, only Ara h 7.0201 was identified at the protein level in peanut protein extracts. It is a 17.3-kDa protein with a pI of 7.7 and makes up only 0.5 % of total peanut protein extract. Ara h 7.0201 shows 42 % amino acid sequence identity to Ara h 2.0201 and 43 % to Ara h 6, the highest diversity being found in the hypervariable region. Sera of around 43 % of 40 peanut-allergic patients contained IgE specific for Ara h 7.0101, but no study on the prevalence or allergenic properties has been performed for Ara h 7.0201 which is the Ara h 7 isoform that is actually present in peanut [79].

### The nsLTP Ara h 9

The nsLTPs share a common architecture that is characterized by the presence of a hydrophobic cavity enclosed by four or five alpha helices and stabilized by four conserved disulfide bridges [61]. Sequence identities between nsLTPs from different plants tend to be low even though their 3-dimensional structures are very similar. Although their ability to transfer various lipid molecules between lipid bilayers in vitro has been well reported, the biological function of nsLTPs in vivo is still unclear. Their involvement in plant defense against pathogens and in the formation of protective hydrophobic layers on the surface of some plant organs has been suggested (reviewed in [10•, 80]).

Ara h 9 is a typical nsLTP with a molecular mass of 9.1 kDa and a basic pI of 9.2–9.4. Two isoforms of Ara h 9 with a sequence identity of 90 % were designated as Ara h 9.0101 and Ara h 9.0201. Many studies have reported the importance of Ara h 9 in peanut-allergic patients, mainly in the Mediterranean area but also in some nsLTP-sensitized patients from Central Europe [81, 82, 83•]. Recently, a report was published about the strong association between peach, peanut, and hazelnut allergy in a Spanish peanut-allergic patients group [84]. Ninety percent of 42 peanut-allergic patients were sensitized to Pru p 3, 82 % to Ara h 9, and 74 % to the hazelnut nsLTP Cor a 8. Most of the patients reported that symptoms to peanut and later on to hazelnut appeared after they had become allergic to peach. Pru p 3 showed a strong capacity to inhibit IgE-binding to Ara h 9 and Cor a 8, while Ara h 9 and Cor a 8 were unable to inhibit IgE-binding to Pru p 3. Altogether, these findings suggested that the peach allergen Pru p 3 in these patients acted as the primary sensitizer [84].

## The Peanut Profilin Ara h 5

The peanut profilin, Ara h 5, is a minor peanut allergen which is involved in pollen-associated peanut allergy. Different profiles of profilin sensitization in peanut-allergic patients have been described in different areas of the world, with a sensitization rate of 3.3 % in the United States, 9–16 % in Northern and Central Europe, and 24 % in Spanish peanut-allergic patients [17, 83•, 85, 86]. This is in line with the assumption that, in accordance with the prevalence of sensitization to birch and grass pollen in Northern, Central and Southern Europe, primary sensitization to profilin is caused by birch pollen profilin Bet v 2 and/or grass pollen profilin Phl p 12. This is also supported by the study of Cabanos and colleagues where IgE reactivity to Ara h 5 coincided with that of two other profilins, Phl p 12 and Bet v 2, confirming cross-reactivity [85]. They also found potential surface-exposed sequential and discontinuous epitopes of Ara h 5, comprising highly conserved and variable regions, features that may contribute to the cross-reactivity as well as species-specific IgE-reactivity. The presence of the putative specific epitopes might explain the relatively higher IgE-reactivity of the three peanut-allergic patients to Ara h 5 as compared to Phl p 12 and Bet v 2.

Wang and colleagues have recently solved the structure of Ara h 5 (PDB:4ESP) and demonstrated that the overall fold of the protein, similar to previously described profilins, had a central seven-stranded antiparallel β-sheet with two α-helices at the amino-terminal side on one side and one helix at the carboxy-terminal side [87] (Fig. 2). Structure alignments revealed that Ara h 5 is more similar to Bet v 2 than to Hev b 8, although sequence alignments suggested that Ara h 5 is more closely related to Hev b 8 than to Bet v 2.

## The Bet v 1-Like Ara h 8

Bet v 1 from birch pollen often induces cross-reactive IgE that reacts with related allergens in certain fruits, vegetables, tree nuts and legumes including peanut. Peanuts contain an allergenic member of the family 10 of pathogenesis-related proteins, the Bet v 1-related allergen Ara h 8. Mittag and colleagues cloned its cDNA, expressed and characterized the recombinant protein [88]. Of the 20 peanut- and birch pollen-allergic individuals included in this study, 17 had IgE specific for rAra h 8. In 12 of these 17 patients, the anti-peanut response was dominated by Ara h 8. IgE binding to rAra h 8 was inhibited by Bet v 1 in peanut extract immunoblotting and in RAST inhibition. On DBPCFC, two of three Ara h 8-monosensitized patients experienced systemic reactions whereas one patient had only OAS. These data showed that a clinically relevant cross-sensitization, and the risk to develop serious allergic reactions existed in individuals with concurrent birch pollen and peanut allergy. Potential cross-reactivity between Ara h 8, Bet v 1 and a PR-10 protein from white lupin was shown by an in silico approach [89]. Cross-reactivities between rBet v 1, rAra h 8 and rGly m 4, the homolog from soybean, were later confirmed by performing IgE inhibition studies with sera of birch pollen-allergic patients who also had food allergy to peanut and soy [90]. In the same study, one IgE-binding surface area present on all three molecules was identified by using phage-displayed epitope mimics and computer-based mapping of the selected peptide mimics. Riecken and colleagues developed a purification strategy for natural Ara h 8 from peanuts and could show that the purified protein, designated as Ara h 8.0201, differed significantly from the previously published Ara h 8.0101 isoform whose sequence they could only identify on the genomic DNA but not the mRNA level [91]. The authors emphasize that the natural counterpart to a recombinant allergen represents an excellent and necessary reference point. Very recently, the structure of the Ara h 8.0101 isoform was determined from a recombinant protein expressed in *E. coli* [92•] (Fig. 2). The authors surveyed an array of potential physiologically relevant ligands and identified the phytoestrogen flavonoids quercetin, apigenin, and daidzein as avidly binding in the hydrophobic cavity of Ara h 8.0101, suggesting that Ara h 8 might serve as a delivery vehicle for flavonoids. Quercetin-3-O-sophoroside was identified as the natural ligand of Bet v 1 [93]. The IgE reactivity and the proteolytic stability of nAra h 8 was shown to be increased after roasting possibly due to the association of the allergen with lipophilic ligands and/or the formation of neoepitopes [94].

## The Peanut Oleosins Ara h 10 and Ara h 11

Peanut oleosins have also been implicated in peanut hypersensitivity [95]. In oil seeds, oleosins are structural proteins of intracellular lipid storage organelles called oil bodies. Oil bodies include a matrix of triacylglycerols (oil) enclosed by a layer of phospholipids embedded with oleosins. Oleosins are restricted to plants and are very abundant in seeds, e.g., peanuts, due to their high oil contents (44–56 % [1]), but they are also found in the tapetum cells of anthers in *Arabidopsis* and *Brassica*. An oleosin molecule can be divided into the N- and C-terminal hydrophilic portions on the surface of the oil bodies and a central hydrophobic domain that contains the unique proline knot that is anchored into the oil bodies. Interestingly, the central hydrophobic stretch of 72 uninterrupted non-polar residues which penetrates the surface phospholipid monolayer is twice as long as any found in all prokaryotic and eukaryotic proteins (for reviews, see [96, 97]). Recently, it has been shown that oleosins, beside their structural function, may also assist in the biosynthesis and mobilization of oils. The oleosin 3 (OLE3; UniProt: Q647G4) of immature peanut seeds has been shown to have both a monoacylglycerol acyltransferase and phospholipase A2 activity suggesting involvement in accumulation of oil and phospholipid hydrolysis [98].

Although the first formal evidence of an 18 kDa oleosin involvement in allergy was reported more than 10 years ago, data about in vitro and in vivo relevance of oleosins for peanut-allergic patients are very scarce. Up to now, five different IgE-binding peanut oleosins with a molecular weight from 14–18 kDa and alkaline pI from 8.9–9.6 have been identified [95, 99, 100]. Two of them are included in the official list of allergens. A 14-kDa peanut oleosin was designated as Ara h 11 (UniProt No. Q84T21) and the two isoforms with a molecular weight of 17.8 and 15.5 kDa and a sequence identity of 87 % were designed as Ara h 10.0101 (UniProt No. Q647G5) and Ara h 10.0102 (UniProt No. Q6474). The sequence identity between Ara h 11 and the two Ara h 10 isoforms is only 30 %. According to www.allergen.org, both Ara h 10 and Ara h 11 were recognized by IgE of 7 peanut-allergic patients as determined by Blot/CAP performed with a purified natural oleosin fraction obtained from peanut oil bodies. An 18-kDa peanut oleosin (UniProt No. Q9AXI1) shows a sequence identity of 57 % to Ara h 10.0101 and 42 % to Ara h 11.

Pons et al. showed specific IgE-binding to this 18-kDa oleosin isolated from purified peanut oil bodies in 3 of 14 sera of patients allergic to peanut [95]. IgE reactivity of the 18 kDa oleosin increased when roasting peanuts. The oleosin monomer displayed only weak IgE-binding but strong IgE-binding to its oligomers of 34, 50, and 60 kDa was observed. The authors also point to a possible cross-reactivity between peanut and soy, as the two sera with peanut oleosin-specific IgE also reacted with soy oleosins. The cross-reactivity of peanut and soy oleosins is based on their high sequence identity ranging from 50 to 79 %.

The most recently reported IgE-binding peanut oleosin, oleosin 3 (UniProt No. Q647G3), is a 16.8-kDa molecule and shows only up to 41 % identity to the other three oleosins [99]. An oleosine 3 peptide derived from α-chemotryptic hydrolysate of total peanut proteins appears to be an IgE epitope cross-reacting with buckwheat. The peptide identified as SDQTRTGY is identical to amino acids 2–9 in the N-terminal hydrophilic domain of oleosin 3 and was found to bind IgE of all 8 tested peanut-allergic sera. The peptide inhibited IgE-binding to the PBS-soluble fraction of buckwheat in a range of 10–50 % using sera of 7 peanut-allergic patients.

Oleosins have also been identified as allergens in two other seeds, namely sesame [101] and hazelnut [102]. Ara h 10 isoforms show a higher sequence identity to the hazelnut oleosin Cor a 12 (56 %) and the sesame oleosin Ses i 4 (42 %), while Ara h 11 shows higher sequence identity to Cor a 13 (69 %) and Ses i 5 (75 %). Nevertheless, the clinical relevance and IgE cross-reactivity of these allergens are unknown and requires more research.

## The Peanut Defensins Ara h 12 and Ara h 13

Plant defensins are small, highly stable, cysteine-rich peptides that are a part of the innate immune system and that have antifungal, antibacterial, protease inhibitory, or insect amylase inhibitory activity [103]. Ara h 12 and Ara h 13 are peanut defensins that were isolated by methanol/chloroform extraction from ground peanuts [104]. IgE reactivity was shown for a small number of sera with severe peanut allergy. As peanut defensins have only low sequence identity with the allergenic defensins from soybean (Gly m 2) and mugwort pollen (Art v 1), cross-reactivity is not to be expected.

## Cross-Reactivity of Peanut Allergens with Lupin Allergens

The interest to exploit alternative vegetable protein sources to soy has led to an increasing use of sweet lupin seed flour in bakery, confectionary, snack, and pastry products as an additive to wheat flour or as a substitute for soy flour. Lupin allergy seems to appear in patients with an existing peanut allergy but can also manifest in isolation [105, 106]. The majority of lupin seed proteins are comprised of α-conglutins (legumin-like) and β-conglutins (vicilin-like), and to a lesser extent γ-conglutins (vicilin-like) and δ-conglutins (2S albumins). Sera from six patients with a positive double-blind, placebo-controlled food challenge to lupin protein-containing food preparations and a coexisting peanut allergy were used to analyze the cross-reactivity of legume seed storage proteins [107]. While all sera reacted with the legumin-like α-conglutin fraction, IgE from sera of five of the six study individuals bound to the vicilin-like β-conglutins. In an indirect ELISA, peanut and almond protein extracts were able to inhibit IgE-binding to the lupin protein extract. As individual lupin conglutin fractions were not coated to the plates, the extent of inhibition by Ara h 1 or Ara h 3 was not determined. In a follow-up study again using six lupin allergic patients’ sera, the inhibitory capacity of Ara h 1, Ara h 2, and Ara h 3 on IgE binding to lupin conglutins was determined [108]. While Ara h 1 most potently cross-reacted with β-conglutin and Ara h 2 with δ-conglutin, Ara h 3 only displayed inhibition of IgE binding to α-conglutin at highest concentrations. It is worth to note that the 2S albumin Ara h 2 showed the strongest inhibition of the legumin-like α-conglutin indicating a cross-reactivity between unrelated allergens, as has been described by Bublin and colleagues for peanut [11•].

A β-conglutin from *L. angustifolius* is included as the allergen Lup an 1 in the official allergen nomenclature database [109]. Another lupin vicilin-like protein with IgE-binding capacity from *L. albus* named Lup-1 showed around 40 % sequence identity to related allergens from peanut (Ara h 1), pea (Pis s 1), and lentil (Len c 1) [110]. A recent study recruited 12 peanut-allergic children to identify the lupin allergens responsible for cross-reactivity with peanut [111]. The results suggested the β-conglutin Lup an 1 as the major cross-reactive lupin allergen having been recognized by cutaneous IgE in 7/12 patients. In skin prick tests, 4/12 patients tested positive to γ-conglutin, 5/12 to α-conglutin, and 3/12 to δ-conglutin.

In skin prick tests performed on ten well-characterized peanut-allergic patients, seeds from eight different legume species produced positive results [112]. In a Western blot, sera from a subgroup of these patients showed IgE binding to vicilins from soybean, pea, and lupine seeds. Ballabio and colleagues studied the cross-reactivity of peanut allergens and proteins from lupin, lentil, pea, kidney bean, and soybean in 12 peanut-allergic subjects [113]. Using a combination of immunoblotting, N-terminal sequencing, and skin prick testing, the basic subunits of the 11S globulins were identified as the most cross-reactive polypeptides in this patient cohort.

## Conclusions

Peanut allergens such as the Bet v 1-related Ara h 8, the peanut profilin Ara h 5, and the nsLTP Ara h 9 are types of allergens that are considered as panallergens. Such allergens are responsible for allergic cross-reactivities across a wide variety of unrelated plants and are often associated with birch and grass pollinosis or involved in the so-called nsLTP-syndrome. On the other hand, the vicilin Ara h 1, the 2S albumins Ara h 2 and Ara h 6, and the legumin Ara h 3 represent major constituents of the peanut that frequently bind IgE. Co-sensitization to these allergens seems to be predictive of more severe reactions. Among these, Ara h 2 has been identified as an important predictor of clinical reactivity to peanut. Together with Ara h 6, Ara h 2 accounted for the majority of the effector activity in peanut extracts, and the two allergens were the major elicitors of anaphylaxis in peanut-allergic mice. Thus, Zhuang and Dreskin suggested a redefiniton of the major peanut allergens with only Ara h 2 and Ara h 6 to be termed major allergens [114•].

In our recent study [11•], we found that the members of two unrelated protein families, the 2S albumins and the cupins, are highly cross-reactive in peanut. This is due to the presence of highly similar sequence stretches present on surface-exposed loops. IgE cross-reactive between Ara h 2, Ara h 1, and Ara h 3 comprises the major fraction of IgE-specific for these allergens. It also has the highest affinity to Ara h 2. Likewise, other researchers observed cross-reactivates between unrelated allergens, indicting that simple comparison of amino acid sequences may be insufficient to predict cross-reactivity. The presence of IgE cross-reactivity between peanut allergens and allergens from other legumes and tree nuts has been demonstrated in some studies, but the identification of the involved individual allergens is still limited. Such studies are needed to answer the question whether allergic reactions to legumes and tree nuts in peanut-allergic individuals are a result of a primary allergy to peanut or cross-reactivity.
